# National Institutes of Health Funding for RNA Vaccine Research

**DOI:** 10.1001/jamanetworkopen.2026.0046

**Published:** 2026-03-02

**Authors:** Anirudha S. Chandrabhatla, Adishesh K. Narahari, Kevin Jin, Benjamin Mazurek, Taison D. Bell

**Affiliations:** 1 Feinberg School of Medicine, Northwestern University, Chicago, Illinois; 2 School of Medicine, University of Virginia, Charlottesville

## Abstract

This cross-sectional study describes the state of National Institutes of Health funding for research related to RNA vaccines.

## Introduction

National Institutes of Health (NIH) funding is critical for biomedical research. There has been increased scrutiny on funding for RNA-based vaccine research, potentially threatening decades of progress. We assessed the state of NIH funding for research related to RNA vaccines.

## Methods

This cross-sectional study was deemed exempt from institutional review board review and informed consent by the University of Virginia because the study solely uses publicly available data. This study is reported following the Strengthening the Reporting of Observational Studies in Epidemiology (STROBE) reporting guideline for cross-sectional studies.

NIH Research Portfolio Online Reporting Tools Expenditures and Results was queried for active extramural grants with the search term *RNA* and *vaccine* or *RNA vaccine* or *RNA-vaccine*. Grants were reviewed to ensure relevance to RNA vaccines. The following were collected for each grant: grant number, title, institution, funding, funding NIH institution, publications, citations, and publication distribution across human, animal, and cell or molecular.^1,2^ Grants were categorized into study area based on project abstracts. Analyses were conducted using Python version 3.14 (Python Software Foundation) from August to December 2025.

## Results

A total of 178 active NIH grants with start years between 1997 and 2025 were included, accounting for $1.65 billion in funding. R01s were the most common (67 grants; $139 million) (Table 1), followed by R21s (26 grants; $11 million), and R43s (11 grants; $4 million). The National Institute of Allergy and Infectious Diseases was the most common awarding institute (110 grants; $1.08 billion), followed by the National Cancer Institute (27 grants; $512 million), and the National Institute of General Medical Sciences (10 grants; $5.5 million). The top 3 areas of study by grants and funding were viruses (75 grants; $968 million), RNA technology (46 grants; $512 million), and cancer (19 grants; $75 million) (Table 2). The grants produced 2342 publications and 149 033 citations. Publications spanned the cell, animal, and human research spectrum (Table 2). There were no patents linked to the grants. Overall, 236 publications (11%) were clinical trials or practice guidelines. In addition, 787 publications (35%) were cited 5949 times in clinical trials or practice guidelines. Of 75 grants studying viruses, 71% were related to either COVID-19 (29 grants) or HIV (24 grants) (Table 2). Grant funding was distributed across the US. The top 3 states in terms of funding were Washington (16 grants; $558 million), Maryland (6 grants; $407 million), and North Carolina (13 grants; $226 million). In total, 18 grants were awarded to 15 unique small businesses across genomics, biomaterials, and chemical manufacturing through the Small Business Innovation Research and Small Business Technology Transfer mechanisms.

**Table 1.** Breakdown of Grants by Grant Type

Category	Grants, No.	Grant type, No.						
		U	R (Non-R01)	R01	P	K	F	Other
Viruses								
COVID-19	29	5	7	13	0	1	1	2
HIV	24	3	7	8	3	1	0	2
Influenza	6	1	4	1	0	0	0	0
Hepatitis	2	0	0	2	0	0	0	0
Influenza and tuberculosis	1	0	0	0	0	0	0	1
Zika	1	0	0	1	0	0	0	0
Paramyxoviruses and bunyaviruses	1	1	0	0	0	0	0	0
Dengue	1	0	0	1	0	0	0	0
Chikungunya	1	0	1	0	0	0	0	0
Dengue and zika	1	0	0	1	0	0	0	0
Orthobunya	1	0	1	0	0	0	0	0
Phenuiviruses	1	1	0	0	0	0	0	0
Coxsackie	1	0	1	0	0	0	0	0
Respiratory viruses	1	0	0	1	0	0	0	0
RSV and hMPV	1	0	0	1	0	0	0	0
Beta coronavirus	1	0	0	0	1	0	0	0
Coronavirus (general)	1	0	0	0	1	0	0	0
HSV	1	0	0	1	0	0	0	0
RNA technology	46	2	20	15	1	2	1	5
Cancer								
Solid tumors	8	1	1	3	0	1	2	0
Melanoma	3	0	0	0	0	0	0	0
Cancer (general)	2	0	1	1	0	0	0	0
Pancreatic cancer	1	0	1	0	0	0	0	0
Kaposi sarcoma	1	1	0	0	0	0	0	0
Lung and prostate cancer	1	0	0	1	0	0	0	0
Sarcoma	1	0	0	0	1	0	0	0
Breast cancer	1	1	0	0	0	0	0	0
Myeloma	1	0	0	0	1	0	0	0
Other	0	0	0	0	0	0	0	0
Tick-borne illnesses	5	0	4	0	1	0	0	0
Cardiovascular	2	0	0	2	0	0	0	0
Lymphangioleiomyomatosis	1	0	0	1	0	0	0	0
Alzheimer	1	0	0	1	0	0	0	0
Retinal disease	1	0	1	0	0	0	0	0
Allergy	1	0	1	0	0	0	0	0
ARDS	1	0	0	1	0	0	0	0
DVT	1	0	0	0	0	1	0	0
Bacteria								
Tuberculosis	4	0	3	1	0	0	0	0
* Staphylococcus aureus*	1	0	0	0	1	0	0	0
Salmonella	1	0	1	0	0	0	0	0
Lyme	1	0	0	1	0	0	0	0
Syphilis	1	1	0	0	0	0	0	0
Nontuberculous mycobacteria	1	0	0	1	0	0	0	0
Group A *Streptococcus*	1	0	0	1	0	0	0	0
Pertussis	1	0	0	1	0	0	0	0
Parasite								
Malaria	6	0	2	3	0	1	0	0
* Toxoplasma gondii*	1	0	1	0	0	0	0	0
Schistosomiasis	1	0	1	0	0	0	0	0
Leishmaniasis	1	0	0	1	0	0	0	0
* Opisthorchis viverrini*	1	0	0	1	0	0	0	0
Fungi								
* Cryptococcus*	2	0	1	1	0	0	0	0
* Candida*	1	0	0	0	0	1	0	0
Coccidioidomycosis	1	1	0	0	0	0	0	0

Abbreviations: ARDS, acute respiratory distress syndrome; DVT, deep vein thrombosis; hMPV, Human metapneumovirus; HSV, herpes simplex virus; RSV, respiratory syncytial virus.

**Table 2.** RNA Vaccine Grants Stratified by Area of Study

Category	Grants, No.	Funding, $	Publications, No.	Citations, No.	Publications, No.		
					Human	Animal	Cell or molecular
Viruses							
Overall	75	967 910 599	921	64 294	473	132	316
COVID-19	29	510 221 666	578	46 456	368	26	184
HIV	24	172 558 770	159	9114	58	44	57
Influenza	6	11 122 504	62	5340	26	10	26
Hepatitis	2	3 396 620	5	44	1	3	1
Influenza and tuberculosis	1	1 989 733	0	0	NA	NA	NA
Zika	1	1 768 228	1	0	0	1	0
Paramyxoviruses and bunyaviruses	1	82 150 482	5	10	3	0	2
Dengue	1	2 268 440	10	286	0	9	1
Chikungunya	1	599 938	0	0	NA	NA	NA
Dengue and zika	1	3 590 945	6	69	0	3	3
Orthobunya	1	468 636	5	15	0	2	3
Phenuiviruses	1	88 217 304	3	5	2	0	1
Coxsackie	1	433 175	0	0	NA	NA	NA
Respiratory viruses	1	2 120 971	1	6	0	1	0
RSV and hMPV	1	2 142 166	1	18	1	0	0
Beta coronavirus	1	55 999 996	48	2407	8	20	20
Coronavirus (general)	1	23 236 986	18	135	2	5	11
HSV	1	5 624 039	19	389	4	8	7
RNA technology	46	511 826 431	641	43 209	339^a^	132^a^	192^a^
Cancer							
Overall	19	75 051 791	482	34 242	339	41	102
Solid tumors	8	14 264 322	26	292	11	5	10
Melanoma	3	2 141 708	11	65	3	6	2
Cancer (general)	2	2 076 954	5	16	1	1	3
Pancreatic cancer	1	408 331	6	43	3	0	3
Kaposi sarcoma	1	2 397 203	2	0	0	1	1
Lung and prostate cancer	1	2 660 172	14	317	4	1	9
Sarcoma	1	19 509 478	252	9203	208^a^	11^a^	32^a^
Breast cancer	1	597 289	0	0	NA	NA	NA
Myeloma	1	30 996 334	168	24 306	109^a^	16^a^	42^a^
Other							
Overall	13	28 268 022	79	1178	2	67	10
Tick-borne illnesses	5	20 840 310	68	1049	2	58	8
Cardiovascular	2	2 322 704	1	0	0	1	0
Lymphangioleiomyomatosis	1	1 305 047	1	0	0	0	1
Alzheimer	1	835 417	0	0	NA	NA	NA
Retinal disease	1	294 052	0	0	NA	NA	NA
Allergy	1	461 270	0	0	NA	NA	NA
ARDS	1	2 057 699	9	129	0	8	1
DVT	1	151 523	0	0	NA	NA	NA
Bacteria							
Overall	11	40 699 227	90	2622	27	36	27
Tuberculosis	4	6 658 815	2	25	1	0	1
* Staphylococcus aureus*	1	19 808 108	61	2412	25	16	20
Salmonella	1	225 839	2	3	0	1	1
Lyme	1	3 240 479	10	107	1	8	1
Syphilis	1	4 663 782	4	11	0	2	2
Nontuberculous mycobacteria	1	950 092	0	0	NA	NA	NA
Group A *Streptococcus*	1	1 030 794	0	0	NA	NA	NA
Pertussis	1	4 121 318	11	64	0	9	2
Parasite							
Overall	10	10 708 200	82	2930	17	50	15
Malaria	6	4 925 109	1	0	0	1	0
* Toxoplasma gondii*	1	457 123	1	3	0	1	0
Schistosomiasis	1	300 000	0	0	NA	NA	NA
Leishmaniasis	1	769 366	1	5	0	1	0
* Opisthorchis viverrini*	1	4 256 602	79	2922	17	47	15
Fungi							
Overall	4	15 005 207	47	558	5	36	6
* Cryptococcus*	2	3 879 407	30	465	4^a^	22^a^	4^a^
* Candida*	1	269 800	4	3	0	4	0
Coccidioidomycosis	1	10 856 000	13	90	1	10	2
Total	178	1 649 469 477	2342	149 033	1180	494	668

Abbreviations: ARDS, acute respiratory distress syndrome; DVT, deep vein thrombosis; hMPV, human metapneumovirus; HSV, herpes simplex virus; NA, not applicable; RSV, respiratory syncytial virus.

^a^ Data for 1 publication missing in the National Institutes of Health database.

## Discussion

In this cross-sectional study, we describe the state of NIH funding for RNA vaccine research in a time of scrutiny on this science. Most grants were studying vaccines for viruses, although there was significant investment to develop RNA technology in general and vaccines for cancer and bacterial, fungal, and parasitic infections.

COVID-19–related research accounted for 29 of 75 grants studying viral vaccines, reflecting the prioritization of this research during the pandemic.^3^ Still, the approximately $510 million funding in these grants was much less than the tens of billions estimated to have been spent on COVID-19 hospitalizations.^4,5^ We found that NIH funding supported other high-impact applications of RNA vaccines, from HIV and hepatitis to highly contagious tropical viruses. Our analyses also revealed considerable efforts studying RNA vaccines for noninfectious diseases. Oncology was the most prominent example, with grants spanning solid and hematologic malignant neoplasms. RNA vaccines for cancer represent a novel frontier that could be paradigm-shifting, with multiple promising phase 1 and 2 trials.^6^

The grants we analyzed have resulted in strong scientific output, with more than 2300 publications and nearly 150 000 citations across basic, translational, and clinical research. The clinical impact of this work was apparent, with 10% of publications classified as and 35% being cited in clinical trials or practice guidelines. Even more, 18 grants through Small Business Innovation Research and Small Business Technology Transfer underscores how this funding supports biotech entrepreneurship.

RNA vaccine research has implications for millions of people in the US and billions worldwide. Our findings underscore the role of NIH funding in shaping RNA vaccine technology beyond COVID-19 to include other infectious and chronic diseases. We did not evaluate non-NIH federal funding and could not link funding explicitly to specific therapeutics on the market. Funding decisions for RNA vaccine research needs scrutiny before potentially eliminating decades of progress.

## Notes

Supplement. Data Sharing Statement
