# Supplementary material for: National Institutes of Health Funding for RNA Vaccine Research
Source: JAMA Netw Open. 2026 Mar 2;9(3):e260046. doi: 10.1001/jamanetworkopen.2026.0046 (PMC12954538; doi:10.1001/jamanetworkopen.2026.0046)
Supplement: Supplement. — Data Sharing Statement [file jamanetwopen-e260046-s001.pdf]

Data Sharing Statement

Chandrabhatla. National Institutes of Health Funding for RNA Vaccine Research. *JAMA Netw Open*. Published March 02, 2026. doi:10.1001/jamanetworkopen.2026.0046

Data
Data available: Yes
Data types: Data (not involving human participants)
How to access data: Data can be accessed online or by emailing the corresponding author.
When available: With publication

Supporting Documents
Document types: Statistical/analytic code
How to access documents: Emailing the corresponding author.
When available: With publication

Additional Information
Who can access the data: Emailing the corresponding author.
Types of analyses: For academic research.
Mechanisms of data availability: Emailing the corresponding author.
